# Devastated Bladder Outlet in Pelvic Cancer Survivors: Issues on Surgical Reconstruction and Quality of Life

**DOI:** 10.3390/jcm10214920

**Published:** 2021-10-24

**Authors:** Francisco E. Martins, Henriette Veiby Holm, Nicolaas Lumen

**Affiliations:** 1 Department of Urology, School of Medicine, University of Lisbon, Hospital Santa Maria/CHULN, 1649-035 Lisbon, Portugal; 2 Department of Urology, Oslo University Hospital, N-0424 Oslo, Norway; holm.henriette@gmail.com; 3 Department of Urology, Ghent University Hospital, 9000 Ghent, Belgium; nicolaas.lumen@uzgent.be

**Keywords:** devastated bladder outlet, posterior urethral stenosis, bladder neck contracture, vesicourethral anastomotic stenosis, prostate cancer, radiation therapy, radical prostatectomy, adverse effects, reconstruction

## Abstract

Bladder outlet obstruction following treatment of pelvic cancer, predominantly prostate cancer, occurs in 1–8% of patients. The high incidence of prostate cancer combined with the long-life expectancy after treatment has increased concerns with cancer survivorship care. However, despite increased oncological cure rates, these adverse events do occur, compromising patients’ quality of life. Non-traumatic obstruction of the posterior urethra and bladder neck include membranous and prostatic urethral stenosis and bladder neck stenosis (also known as contracture). The devastated bladder outlet can result from benign conditions, such as neurogenic dysfunction, trauma, iatrogenic causes, or more frequently from complications of oncologic treatment, such as prostate, bladder and rectum. Most posterior urethral stenoses may respond to endoluminal treatments such as dilatation, direct vision internal urethrotomy, and occasionally urethral stents. Although surgical reconstruction offers the best chance of durable success, these reconstructive options are fraught with severe complications and, therefore, are far from being ideal. In patients with prior RT, failed reconstruction, densely fibrotic and/or necrotic and calcified posterior urethra, refractory incontinence or severe comorbidities, reconstruction may not be either feasible or recommended. In these cases, urinary diversion with or without cystectomy is usually required. This review aims to discuss the diagnostic evaluation and treatment options for patients with bladder outlet obstruction with a special emphasis on patients unsuitable for reconstruction of the posterior urethra and requiring urinary diversion.

## 1. Introduction and Terminology

Devastated bladder outlet (including bladder neck and posterior urethra) is defined as an entity associated with refractory, recalcitrant stenosis, significant necrosis, and/or end-stage urinary incontinence, that is deemed unfeasible for reconstruction [1]. It can originate from neurogenic dysfunction, external trauma, or more often from complications of pelvic cancer treatments, predominantly prostate cancer.

To avoid confusion, it is highly recommended to follow the terminology proposed by SIU/ICUD (Societé International d’Urologie/International Consultation on Urologic Diseases), which reviewed the nomenclature for urethral stenosis, stricture, and pelvic fracture urethral injury. The committee determined that urethral terminology should be anatomical and that the preferred term to define urethral narrowing/obliteration is stricture. The term stricture should be reserved only to urethral segments that are surrounded by corpus spongiosum. The term stenosis should be used for narrowing of the bladder neck, prostatic urethra, and membranous urethra, as they are not involved by the corpus spongiosum [2].

Posterior urethral stenosis (PUS) is an encompassing term describing a narrowing from the distal bladder neck to the proximal bulbar urethra, which is based on anatomical location and presence/absence of the prostate. When the prostate is present, PUS can be classified as bladder neck stenosis or contracture, prostatic urethral stenosis, and membranous/bulbomembranous urethral stenosis. The term vesicourethral anastomotic stenosis (VUAS) should be reserved for posterior urethral stenosis that usually occurs at the anastomosis after prostatectomy. Unfortunately, although this SIU/ICUD terminology should be preferred, the literature continues to routinely use bladder neck contracture for vesicourethral anastomotic stenosis, as well as stricture, stenosis, and contracture interchangeably [3]. The vesicourethral anastomosis is the most prevalent location of stenosis following radical prostatectomy, and the bulbomembranous urethra is the location typically affected by radiation therapy [4]. Bladder outlet obstruction (BOO) is a non-specific term covering all of the above.

Adverse urinary effects after prostate cancer treatments include chronic pelvic pain, radiation cystitis, urinary incontinence, urinary fistula (e.g., enterourinary, urosymphyseal), and bladder outlet obstruction. BOO can be temporary, usually associated with prostatic edema and inflammation in the acute phase of RT, or it can be definitive, which usually occurs after attempted surgery or nonsurgical therapy of the BOO.

Management of VUAS after radical prostatectomy is often simple and can be successfully treated by well-described surgical methods, either minimally invasive endoluminal procedures, or open or robotic reconstructive alternatives. The latter treatment alternatives are usually required in patients with recurrent PUS with a history of RT, which can surely result in devastated bladder outlet. In this scenario of devastated bladder outlet, these unfortunate patients are left with few easy and straightforward options: (1) they can either undergo repeated endoluminal treatments with little hope of long-term success, (2) live with a long-term indwelling catheter, or (3) undergo complex, at times extremely difficult, open/robotic reconstruction. These choices must be weighed in the presence of a good functional bladder and urinary continence status.

This review aims to discuss the diagnostic evaluation and treatment options for BOO with a special emphasis on patients unsuitable for reconstruction of the posterior urethra and requiring urinary diversion.

We present this article as a narrative review. The literature search involved the keywords mentioned above and the databases PubMed, Scopus, and Google Scholar where searched. The most relevant literature currently available and dating back to 2000 and up to the present date was included, except for a few relevant studies published prior to this time limit. Single case reports or series with less than 15 patients were excluded.

## 2. Incidence, Etiology, and Epidemiology

The incidence of prostate cancer, and consequently its diagnosis, is on the rise worldwide [5,6]. Additionally, because the 10-year survival for prostate cancer averages 90% (inclusive of all stages, with 99% cancer-specific survival), the potential for long-term morbidity from treatment has also increased up to 5.2% with a higher risk present in those undergoing prostatectomy or BT combined with EBRT [5,6,7]. Fortunately, this resulted in a large percentage of prostate cancer survivors. Sadly, many of these survivors end up suffering from the adverse consequences of the oncologic treatments throughout their long survivorship [8,9]. Robust randomized trials investigating health-related QoL data of longitudinal population-based cohorts have produced relevant information on the accurate impact on functional morbidity resulting from oncologic treatments. A global decline was noted in the long-term functional outcomes at 15 years after primary treatments such as RP or RT [10]. An estimated 90% of these patients developed ED, 18% had UI, and 20–30% of men suffered from bowel urgency and diarrhea. Equally, the Scandinavian Prostate Cancer Group-4 showed in a randomized trial that 84% of men experienced ED, and 41% experienced UI at a median of 12-year follow-up [11].

The detection of the accurate and realistic incidence of PUS and its most severe form, the devastated bladder outlet, resulting from prostate cancer treatments is profoundly dependent on the duration and reliability of long-term follow-up. Unfortunately, follow-up was short in most single institutional series and randomized studies rendering the exact incidence of PUS and devastated bladder outlet difficult to estimate. Jarosek et al. showed that the 10-year cumulative incidence of PUS ranged from 9.6% to 25.9%, according to etiology, i.e., EBRT (9.6%), RP (19.3%), EBRT + BT (19.4%), and RP + EBRT (25.9%) [12]. The merits of this report are a more accurate measure of PUS rates in the community rather than from single institution data, longer follow-up frames, and comparison of the incidence rates after all types of treatment.

Several epidemiological factors are reported to be associated with a higher risk of PUS following prostate cancer treatments, specifically advanced age, which is a known risk factor for complications of the lower urinary tract [11] (Table 1). Similarly, the treatment modality has also shown a solid influence on the chronological occurrence and severity of PUS. VUAS after RP usually occurs in the first 6 post-operative months, while the adverse tissue effects of radiotherapy occur more gradually and are cumulative over time [5]. Interestingly, the international literature reported significant differences in the incidence of VUAS after RP, whether derived from high volume institutions, or from population-based analyses [12,13].

Since the pathogenesis of BOO depends upon the treatment modality utilized, likewise its incidence will also vary. The main obstacle with the studies that evaluate BOO rates is that they only report patients who underwent treatment, thus underestimating the true incidence [14]. Additionally, these rates pertain to different forms of BOO such as VUAS, BNS, BMUS, and prostatic urethral stenosis and not specifically to devastated bladder outlet settings. Studies with short follow-up showed overall post-treatment BOO rates of 5.2% with RP having the highest rate of 8.4% [5]. If these obstruction rates were stratified by therapy modality and ranges, the results were somewhat different (Table 2). The data reported in this study date back to a period when RP was mostly performed open or during the initial phase of robot-assisted RP [15]. Currently, lower rates of BOO have been reported from large volume institutions, such as 2.5% for VUAS [6,16,17,18,19].

Although occurring less frequently than VUAS in the CaPSURE study, rates of irradiation-induced stenosis were reported to rise at the time of latest follow-up, whereas post-surgery BOO plateaued after the initial 6 months of surgery [5].

The rates of BOO caused by EBRT or BT vary widely across the literature. This variation results from RT delivery modality and RT dosage protocol employed. Although current RT protocols globally showed obstruction rates similar to post-RP, combination therapy with EBRT and BT showed the highest rates at 32% after 2 years of follow-up [25]. However, Hindson et al. reported rates from 4% to 9% for combination treatment protocols [26].

Few studies exist reporting long-term outcomes on PUS after focal energy-ablation treatments. A 2% urethral stenosis rate was reported by cryotherapy compared to 0% after HIFU. However, the median follow-up was only 9 months [29]. However, Muto et al. revealed a 6.7% PUS rate after HIFU with a median follow-up of 34 months [30]. Recently, other novel energy-ablative modalities were introduced such as photodynamic therapy, photothermal therapy, and irreversible electroporation. However, the rates of PUS after these modalities are still unknown.

Salvage treatment for prostate tumor recurrence increases the risk for BOO, as it understandably exposes the previously treated tissues to additional injury. In a small series of salvage RP after primary RT, Corcoran et al. reported VUAS rates of up to 40% [23]. However, other authors in a series of close to 200 patients achieved an obstruction rate of 22%, that was, almost half of the rates in the study by Corcoran [31]. The risk for obstruction in patients who received EBRT after RP, as either adjuvant or salvage treatment, ranged between 3% and10% [32,33]. Both salvage cryoablation and salvage HIFU also produced BOO in 5–12% and 15–30% of patients, respectively [27,28,34,35,36]. Salvage treatment protocols also carried a high risk for other severe debilitating complications such as UI, rectourethral, and urosymphyseal fistulae, for which urinary diversion with or without exenteration may be required, as not uncommonly, reconstruction is unfeasible in these settings.

## 3. Pathophysiology

Bladder outlet obstruction that arises after pelvic cancer treatments, predominantly RP or RT/energy-ablative treatments is believed to result from a combination of several treatment and patient factors, such as previous bladder neck or prostate procedures, surgical approaches, severe hemorrhage, significant urinary extravasation, prior radiotherapy, surgeon’s experience, diabetes mellitus, cardiovascular disease, hypertension, smoking, BMI, and age [24]. Borboroglu et al. confirmed the association of the pre-operative conditions associated with peripheral hypovascularity with higher BOO rates. However, this same review did not demonstrate any relationship between factors such as prior TURP, type of anastomotic suture, and duration of post-prostatectomy urethral catheterization, and BNS after RP [37].

### 3.1. Radical Prostatectomy

Several factors are suggested to be in close association with the occurrence of VUAS following RP, such as RP approach and its specific technical points. Types of RP include open (retropubic and perineal), laparoscopic and robot-assisted laparoscopic prostatectomy (RALP). These modalities have had different trends over the last decades. The perineal and laparoscopic approaches have experienced some decline worldwide recently. In contrast, RALP has witnessed a steady increase in many regions of the globe surpassing the once considered gold standard extirpative therapy for localized prostate cancer, radical retropubic prostatectomy. Several studies have reported lower rates of VUAS in population-based groups [38,39,40]. An analysis of the SEER-Medicare database ranging from 2003 to 2007 showed a lower rate of VUAS in the first year following surgery compared to RRP, i.e., 5.8% vs. 14%, respectively [38]. These findings were corroborated by other studies [39,40]. The exact reasons for these remarkably lower rates of VUAS are unclear; however, better mucosa-to-mucosa anastomosis leading to less urine extravasation at the anastomosis, a less hemorrhagic operation, and lack of mucosa eversion [39,41]. Some authors have questioned the role of the urine leak as a causative mechanism of VUAS through an inflammatory reaction, or this leak reflects only evidence of an anastomotic breach, which heals by secondary intention and leads to fibrosis [42].

It is generally agreed that post-RP VUAS occurs in the initial few months after surgery, extremely rarely after the first year, making the treatment rate of VUAS after the initial 12-month post-operative period close to nil [12,17,20]. This finding reflects that VUAS after RP is a peri-operative phenomenon, as opposed to PUS due to RT, which typically occurs several years following the insult.

### 3.2. Radiation Therapy

Primary radiotherapy and RP for localized prostate cancer have, apparently, equivalent oncologic outcomes [43]. Recent literature supports and recommends adjuvant RT to patients at high-risk for local relapse, mostly with positive surgical margins [44,45]. Two distinct mechanisms are responsible for the injury inflicted by RT upon the target tissue: (1) induction of apoptosis and (2) inhibition of mitosis associated with the early generation of highly ROS, rapidly resulting in protein modifications and damage to DNA, RNA, and cell membranes [46]. The long-term effects of the changes induced by RT, specifically fibrosis and scarring, result from poor vascularity associated with chronic endarteritis, fibroblast injury and the deleterious impact on local growth factors and cytokines altering the metabolism of the target tissues [47].

**Figure 1:**
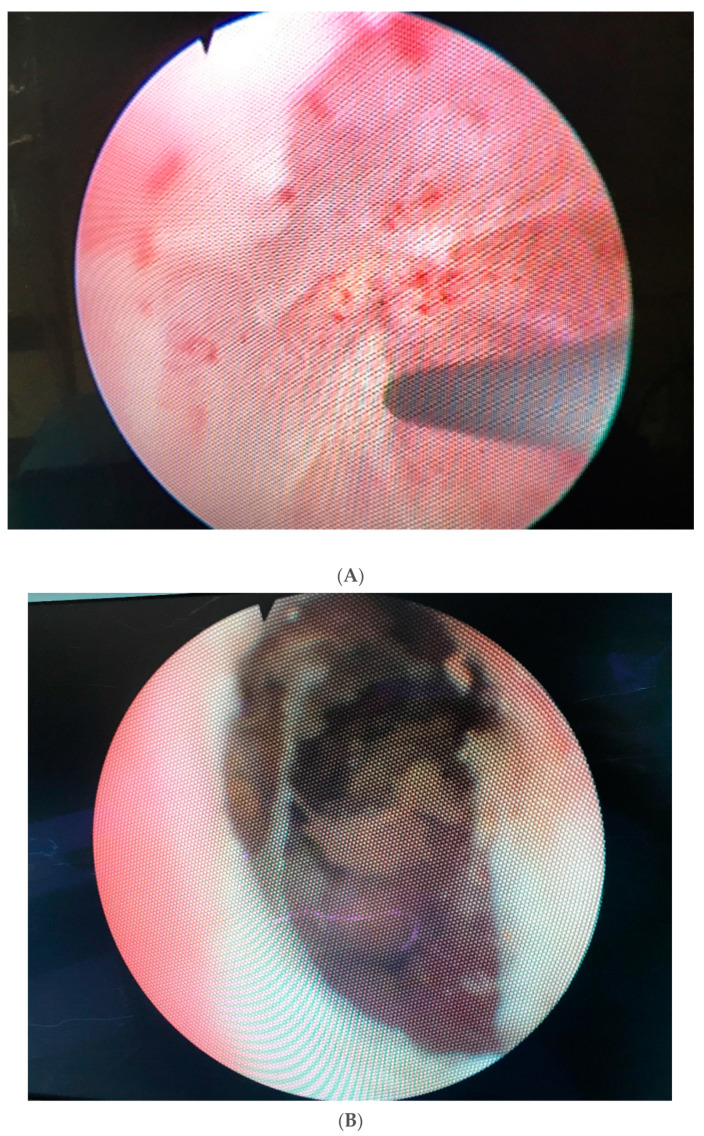
(**A**). Completely obliterated bulbomembranous stenosis (**B**). Fibrotic and necrotic posterior urethral tissue with calcifications caused by radiation.

Radiotherapy employs ionizing radiation to damage the DNA of tumor cells. The ionization process results in the increased production of free radicals, also known as hydroxyl radicals or reactive oxygen species (ROS), which damage the DNA and structural proteins causing cell death [48]. However, the effects of ionization are not limited to tumor cells and can also affect surrounding healthy tissue cells. Collateral damage includes obliterative endarteritis, decreasing tissue blood supply with subsequent hypoxia, and progenitor cell apoptosis. This chain reaction, leading to radiation necrosis, will limit the ability of the involved tissues to heal. RT also produces an accumulation of free radicals and ROS responsible for continuing fibrosis [48]. This progressive tissue scarring will lead to late development of post-RT urethral stenosis, most of which occur up to 3 years after radiotherapy (Figure 1) [20,49,50,51].

RT modality affects the rate and severity of PUS differently. In an analysis of CaPSURE database, Elliott et al. showed a cumulative incidence of PUS requiring treatment at 4 years of 5% for EBRT, 11% for BT, and 16% for combined BT and EBRT [5]. Interestingly and clinically relevant, new adverse events were still arising after RT as opposed to occurring in the first year after surgery. The dose of RT delivered to the bulbomembranous urethra was reported as a significant adverse factor. Consequently, a more precise seed placement at the prostatic apex and a lesser dose of supplemental EBRT has reduced the incidence of PUS following BT [52,53].

Transurethral resection of prostate prior to RT has an increased risk of PUS after RT [54]. In another study, Seymore et al. reported that the incidence of PUS in patients who received RT (either EBRT or BT) after TURP versus patients without previous TURP was 15% and 6%, respectively [55].

Several randomized trials have demonstrated that a higher dose of radiation is linked to better oncological efficacy (translated in lower biochemical recurrence rates) as well as higher adverse effects on surrounding healthy tissues. Research has tried to improve the relation between dose escalation of radiation delivered and simultaneously limiting damage caused on healthy adjacent tissues [56,57]. This concern has led to several approaches including multimodal treatments (BT + EBRT), 3-dimensional conformal RT (3D-CRT), IMRT, and high dose-rate BT. However, these approaches have had different impacts of long-term rectal and urinary toxicities, with IMRT causing lower damage to the rectum [58].

The timing of RT following RP is a major risk factor for the development of PUS. Several randomized as well as population-based studies showed a higher risk of PUS with adjuvant RT as compared to salvage RT [45,59]. This different impact is likely because salvage RT is usually delivered later after surgery than adjuvant RT. The combined therapy with EBRT and high-dose rate BT have shown an exceptionally high stenosis incidence of 32% after a 2-year follow-up [25]. However, more recent protocols have reported stenosis rates between 4% and 9% [26].

Overall, EBRT, BT and cryotherapy usually result in worse PUS compared to RP, which is translated into more common and more invasive treatment modalities needed for the management of PUS after non-surgical therapies compared to PUS after surgery [26,60].

### 3.3. Focal Ablative Therapies

Cryoablation and HIFU are focal, energy ablation treatment modalities developed as alternative ways to treat select patients who wish to avoid major, invasive procedures, and longer hospitalization periods. These therapies may also be used as adjuvant and complementary therapies after RT. Cryotherapy and HIFU also cause destruction of malignant cells through local coagulative necrosis.

Current cryotherapy devices utilize argon and helium gas delivered through needles to produce an ice ball in the targeted tissue. The effect of the fast freezing and cooling of the prostate gland causes cell dehydration and an immediate disruption of the cell membranes due to a mechanical direct effect. The main mechanism of action is by coagulative necrosis, leading to cell death by hypoxic necrosis and apoptosis [21,61]. This process is associated with subsequent prostatic urethral sloughing and dense fibrosis that can lead to severe bladder outlet obstruction [24]. However, this complication decreased with the implementation of urethral warming techniques from 20% to negligible rates [24,62].

**Figure 2:**
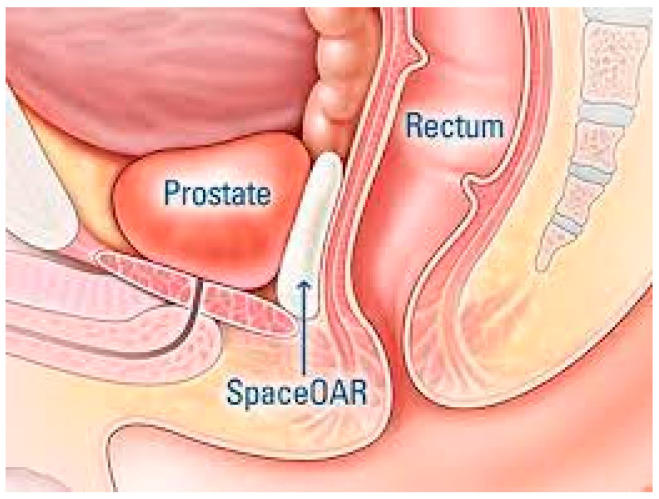
SpaceOAR™ Hydrogel is an absorbable injectable material designed to provide space between the prostate and rectum in men undergoing radiotherapy for pelvic cancers (reproduced from Boston Scientific, Marlborough, MA, USA).

HIFU also has a direct effect on cellular destruction through a coagulative necrosis mechanism using ultrasonic waves at high temperatures that can exceed 100 °C (212 F°) [63]. Unlike cryoablation, HIFU produces tissue necrosis by cavitation of intracellular fluid and destruction by heat of the targeted tissue [64]. Unlike RT, both cryoablation and HIFU do not generate ROS and, therefore, are not associated with progressive stenosis formation. Recently, attempts have been made to reduce the detrimental impact of this progressive stenosis induced by irradiation. In 2015, the Food and Drug Administration cleared SpaceOAR, a novel device to attempt to further reduce toxicity of radiotherapy for prostate cancer (Figure 2) [22].

## 4. Diagnostic Evaluation and Decision Making

Patients presenting with PUS or a devastated bladder outlet after pelvic cancer treatments, may do so early (within 6 months) after surgery or later (>1 year) after RT. When considering surgical treatment BOO, careful evaluation of symptoms is important, as they range from none/few to incapacitating symptoms. However, symptoms of a devastated bladder outlet are usually in the more severe end of the range. Common symptoms are related to lower urinary tract symptoms, including urinary incontinence, bladder dysfunction, incomplete emptying, hematuria, or more severe complications such as rectourethral fistula and total obliteration of vesicourethral anastomosis. Occasionally, the diagnosis is made incidentally when placement of a urethral catheter is attempted for a surgical procedure, i.e., penile prosthesis insertion. It is important to keep in mind that patients with no or few symptoms of BOO following pelvic cancer treatment can be managed conservatively in order not to compromise the often-delicate equilibrium between continence and incontinence in these patients.

Diagnostic evaluation should begin with a detailed history with emphasis on filling and emptying LUTS aided by voiding diaries, validated questionnaires, details of past treatments for pelvic cancer, adjuvant or salvage radiotherapy, and previous interventions for PUS and/or UI [62]. Heavy pelvic irradiation often causes irritative symptoms of varying severity attributable to the location of the damaged tissues and radiation changes to the bladder, bladder neck, and rabdosphincter. Some patients may develop recurrent UTIs (cystitis, prostatitis, and epididymitis), total urinary retention or worsening of renal function. Physical examination is often poor and unrevealing. However, special attention should be directed to the abdominal wall for surgical scars and anomalies of the external genitalia.

Pelvic cancer survivors being considered for elective lower urinary tract surgical reconstruction should undergo routine pre-operative blood tests including renal function parameters, urinalysis, and urine culture. A serum PSA test is critical to exclude prostate cancer recurrence. Urinary cytology should also be included, especially in cases of hematuria, prior history of bladder cancer, and after radiotherapy, known to have an association with metachronous urothelial cancer [65]. Cystourethroscopy gives anatomic evaluation of the location and severity of the obstruction, degree of tissue damage with areas of calcification and necrosis, bladder stones, degree of rabdosphincter function or destruction, and concurrent or recurrent neoplasms. Antegrade cystoscopy should be considered, especially in individuals presenting with a suprapubic catheter, and requiring open surgical reconstruction. This procedure allows a precise description of the proximal extent of the obstruction, which is critical in surgical planning. Some authors also recommend an examination under sedation and cystoscopy as dilatation is often needed [14]. It may assist in a better preoperative assessment of radiation-associated pathology, such as radiation necrosis of the prostate, which is often misinterpreted otherwise. Finally, the appearance of the bladder and bladder neck will assist in determining the salvageability of the lower urinary tract. If poor bladder capacity and compliance is suspected and augmentation cystoplasty is considered, urodynamic evaluation should be performed [66].

Retrograde and voiding cystourethrograms are mandatory in the evaluation of PUS, especially if reconstruction is planned. These studies should complement a full endoscopic evaluation [62]. Although some authors do not formally indicate a CT scan and/or magnetic resonance imaging study of the pelvis, we believe these are crucial in providing relevant information regarding concurrent diagnosis such as fistula formation, prostatic abscess or cavitation, and recurrent malignancy.

## 5. Management

Treatment of bladder outlet obstruction must be individualized and take several critical factors into account such as etiology of the stricture, stricture length and location, health status and wound healing capacity of local tissues, bladder urodynamic features, and continence status to maximize therapeutic success. The SIU/ICUD has recommended an algorithmic approach to the management of BOO [62]. This algorithm has multiple options, ranging from self-dilatation to open surgical reconstruction or urinary diversion. The algorithm for surgical intervention of bladder outlet obstruction can be stratified by severity, length, and location. Short, non-obliterative stenoses are treated with minimally invasive procedures and progressing to more invasive ones for recurrence, while complex stenoses should be initially, and preferentially, approached with open reconstruction, as minimally invasive alternatives are generally doomed to failure.

### 5.1. Endoluminal Management

Several different options have been used for the endoluminal management of BOO. Two different settings should be considered when dealing with patients suffering from BOO or PUS: whether radiation is present or not.

A. VUAS after RP. Endoluminal (dilatation and endoscopic transurethral incision) treatment offers a minimally invasive alternative with an acceptable low complication rate. If the stenosis occurs early as it is common after RP, treatment can be initiated with urethral dilatation, either with sounds, filiforms and followers or coaxial dilators and balloon dilators [67,68]. In the absence of RT, the success rate of dilatation ranges from 40% to 90% after repeated interventions [60,69,70]. Apparently, all these methods share similar success. However, blind procedures, including dilatation, should be avoided in modern times. When available, a guidewire should always be passed across the VUAS first and then proceed with coaxial dilators or balloon dilatation to prevent further urethral or rectal injury. Alternatively, in individuals requiring repeated dilatations or for VUAS occurring at least 2 months after RP, cold knife DVIU should be recommended with success rates ranging from 0% to 90% [37,71,72]. Deep lateral incisions at 4 and 8 o’clock positions are recommended to avoid injury to the neurovascular bundles and rectum. Dorsal incisions should also be avoided not to induce urosymphyseal fistulation [73,74]. If DVIU fails, deep transurethral electrocautery bilateral incision with Collins’s knife should be attempted with success rates reaching 50% in retreatment settings [75]. Patients should be advised that any endoluminal procedure for the treatment of VUAS risks significant injury to the rabdosphincter and consequently UI. Furthermore, patients should also be told that the level of their continence depends on the VUAS and any procedure to widen the stenosis may reveal the true underlying incontinence rather than generating de novo incontinence. If recurrence occurs, these procedures may eventually be potentiated by intralesional injection of antifibrotic agents (e.g., MMC, hyalorunidase, triamcinolone) following an additional incision. The objective of these intralesional injections is to stabilize the fibrotic urethral caliber and consequently to lower the risk of incontinence. Most of these patients have recalcitrant/recurrent non-obliterative VUAS/BNS [76,77]. Reported patency rates with steroid injections range from 50% to 79%, all patients did not require self-dilatation after a maximum of two procedures [78,79,80,81,82,83].

Complications appear to be acceptable across most studies. However, all these studies were retrospective in nature. Redshaw et al. reported Clavien grade 3 complications in 7% (4/56) patients, which included osteitis pubis, rectourethral fistula with trigone necrosis, and severe pain [78]. However, other authors reported more positive results [82,84]. Globally, given the severity of these reported complications, although uncommon, antifibrotic agents, particularly MMC, do need to be used with caution, and preferable inside the framework of a clinical trial [84,85].

Another therapeutic choice for patients with severe refractory VUAS after RP was endoscopic placement of a urethral stent. Urolume (American Medical Systems) was a pioneer for this therapeutic purpose and the first to be approved internationally [86,87]. However, most of its use has been anecdotal [87,88,89]. Patency rates have been relatively modest (47–60%) associated with a high risk of UI [88,89,90]. Complications related to stent migration, tissue ingrowth, and perineal pain restricted its widespread use.

B. PUS after RT. Following RT, patients are more likely to require repeat endoluminal interventions [25]. Irradiation is associated with higher retreatment rates as well as more invasive endoluminal interventions for this pathology. A second dilatation is often used as second procedure for patients after RP, whereas TURP was the second most employed option after irradiation [60]. Radiotherapy is also associated with other more severe complications such as prostatic urethral necrosis, incrustations/calcifications, fistulation (rectal or urosymphyseal), pubis osteitis, cavitation, and abscess formation [4]. If these complications occur, pelvic exenteration and urinary diversion is frequently required [73,91].

### 5.2. Surgical Reconstruction

**Figure 3:**
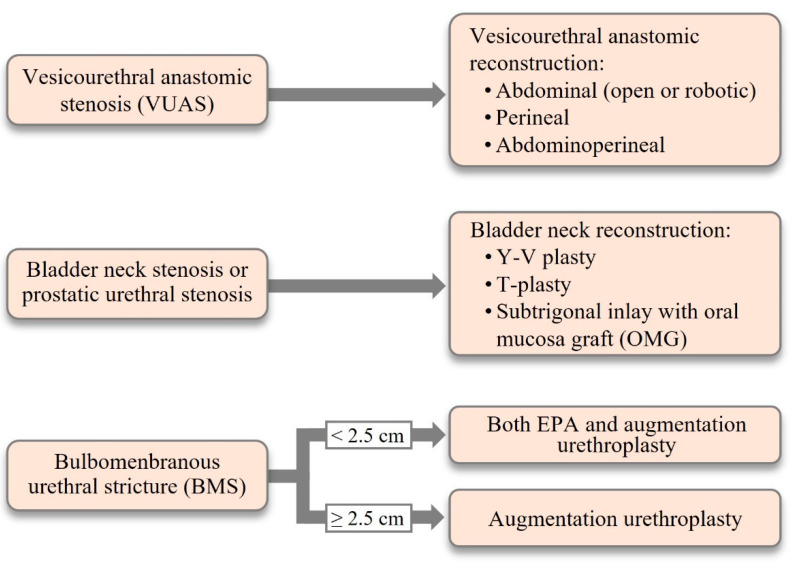
Surgical options for reconstruction of the lower urinary tract due to posterior urethral obstruction in pelvic cancer survivors (Reproduced from EAU Guidelines on Urethral Strictures. European Association of Urology 2021. EAU Guidelines Office, Arnhem, The Netherlands).

A. Bladder outlet obstruction (VUAS) after RP. If endoluminal treatment (persistently) fails or in case of a recalcitrant, totally obliterated posterior urethral stenosis, challenging lower urinary tract reconstruction may be considered in otherwise healthy men motivated to undergo surgery [80,81,92,93]. Open reconstruction will depend upon the length, location, caliber and etiology of the stenosis, continence status, bladder (dys)function, previous radiotherapy, patient’s preference, and surgeon’s expertise (Figure 3). In the authors’ opinion, these complex patients should be referred to high-volume centers specialized in complex urologic reconstruction. Before undertaking open reconstruction, placement of a suprapubic catheter initially can assist with a cystoscopic and imagiologic retrograde/antegrade evaluations (“up-and-down-gram”) that are essential for surgical planning. When possible, video urodynamics is a critical tool to assess bladder (dys)function. Several approaches have been described [66,94,95,96,97]. However, retropubic, transperineal and abdominoperineal, and more recently the robot-assisted, approaches for redo anastomosis are the most frequently employed. The downsides of the retropubic approach include difficult surgical access due to fibrotic obliteration of the Retzius space, compromised visualization, and eventually a short urethral stump. The advantage is the potential to preserve the rabdosphincter. A success rate of 60% with new onset UI of 30% was reported for the retropubic approach [97]. A success rate of 83% was described for the abdominoperineal approach. Noteworthy, all patients in this series received an AUS concomitantly [66]. The transperineal approach has been most widely used due to its lower invasiveness through a potential virgin area, and the possibility of mobilizing the urethra more extensively to bridge any gap created by resection of fibrotic tissue involved in the VUAS. Patency rates varied from 67% to 91% [4,98]. The major disadvantage is damage of the rabdosphincter, making implantation of an AUS universally required [4,98].

Nikolavsky et al. recommended a retropubic approach for VUAS involving the bladder neck, a perineal approach for short VUAS with intact bladder neck and an abdominoperineal approach for long segment (>3 cm) VUAS, including bladder neck involvement [92]. Redo VUA should only be advised in patients with good bladder function and with no (peri)-urethral pathology (urethral necrosis, calcification, fistulation). Flaps (gracilis flap, peritoneal flap, omental flap) to provide support and protection of the anastomosis may be valuable in irradiated patients [92].

B. PUS after RT. Radiation causes stenosis predominantly at the bulbomembranous urethra and differ significantly from those produced by trauma, specifically pelvic fracture injury. Although most of RT-induced stenoses are short, the differences include a severely scarred tissue, deficient wound healing, and less clearly defined tissue planes, making prior RT a risk factor for failure [4,98]. Additionally, bladder toxicity caused by RT may lead to decreased bladder capacity and compliance, bladder spasms and pain, and urethral necrosis making reconstruction futile [91,99]. Therefore, similarly to redo VUA, reconstruction should only be performed in patients with good bladder function and in non-compromised healthy (peri)-urethral tissue. In this setting, flaps (gracilis, peritoneal, or omental) are beneficial in supporting and protecting the in irradiated patients.

The main challenge in the treatment of RT-induced stenosis is further tissue damage with poor healing capacity, involving not only the stenotic area itself but also the adjacent proximal and distal areas of the fibrosis [3,4,62]. Historically, due to these challenges and the likelihood of rabdosphincter involvement, men with RT-induced BMUS have long been regarded less than ideal candidates for urethroplasty and have received urinary diversion if endoluminal modalities failed or were unfeasible. Patency rates of 67% to 95% have been reported for EPA and 50% to 83% for BMG urethroplasty in irradiated patients, with FU ranging from 21 to 66 months [49,50,51,100,101,102]. The continence rates with BMUS urethroplasty, and eventually resection of the rabdosphincter, compared favorably to VUAS reconstruction, likely reflecting an intact bladder neck after RT. If UI occurs following reconstruction of post-RT PUS, it usually results either from previous RT or previous TURP affecting the bladder neck. Additionally, extension of the stenosis through the entire prostatic urethra will require a salvage prostatectomy, including the bladder neck to allow resection of the entire stenotic segment. Hofer et al. achieved 70% patency-free rate with EPA at a mean FU of 3.5 years [49]. However, RT-induced BMUS of more than 2.5 cm are rarely amenable to EPA and are at higher risk of developing de novo UI [103]. De novo UI and ED after urethroplasty for post-RT BMUS are reported to be 11–50% and 0–35%, respectively [49,102,103,104]. Interestingly, augmentation urethroplasty was used in significantly longer strictures compared to those submitted to EPA (respectively 6.1 vs. 2.1 cm; *p* < 0.001). No significant differences in de novo UI (26 vs. 25%; *p* = 1), de novo ED (35 vs. 0%; *p* = 0.06) or other complications (30% vs. 33%; *p* = 1). Hofer et al. reported that 15% of patients treated with EPA needed AUS implantation [49]. These findings emphasize the relevance of pre-operative counseling regarding eventual post-reconstruction UI.

Recently, a “pull-through” procedure has been described as an option to avoid cutaneous diversion in the surgical reconstruction of the devastated PUS associated with a defunctionalized bladder after irradiation where tissue vascularity and quality is severely compromised [42]. This innovative technique of total lower urinary tract reconstruction incorporates salvage cystectomy, ileal neobladder construction, and urethral pull-through followed by AUS implantation at a 2nd stage. All eight patients preserved a functional posterior urethra at a median follow-up of 58 (range 16–84) months. Five (62.5%) patients experienced low-grade complications after the 1st stage, and none developed high-grade complications. Four (50%) patients developed cuff erosion requiring removal and subsequent reinsertion. After a median of two revision surgeries (range 0 to 4), all patients achieved social continence with improvement of QoL [42]. This procedure needs further trials for validation before its use can be advocated.

Radiation therapy and ablation-energy modalities (cryotherapy, HIFU) may cause posterior urethral necrosis, sloughing, cavitation, stenosis, and significant bladder dysfunction making these devastated bladder outlets “non-reconstructable”. These patients as well as patients with significant medical comorbidities are best managed with urinary diversion, mainly if a urethral or suprapubic tube are not tolerated by the patient due to bladder pain or spasms [85,91,99]. Other reasons to abandon the vesicourethral outlet are intractable hematuria and fistulation. Typically, the patient has a history of pelvic RT or ablative interventions for prostate cancer and several previous attempts to achieve cure. Globally, any of the modalities used to treat a devastated posterior urethra depend upon adequate bladder function allowing for bladder sparing as well as healthy intrapelvic ureters [86,92]. In such cases, urinary diversion (continent or incontinent) with or without cystectomy is considered the last resort [99,103]. Cystectomy is performed if palliation of intractable bladder pain, spasms, or hematuria is necessary, which are more prevalent after RT [105,106,107]. Distinct techniques have been reported. However, the selection mostly depends on the bladder function, presence of local symptoms, performance status and patient’s expectations, and surgeon’s training. Most popular options for continent diversion in these individuals are (1) cystectomy and ileocolic (Indiana) pouch or (2) augmentation enterocystoplasty with a catheterizable outlet. Nonetheless, some patients (and urologists) may prefer an ileal conduit to avoid further urinary diversion-related complications and revisions. Any attempt to tunnel a Mitrofanoff or Yang-Monti into an irradiated bladder should be avoided. Sack et al. reported 100% satisfaction rate and most patients would have undergone this extirpative surgery an average of 13 months sooner in a study of 15 patients [108]. In a report by Faris et al., 27% of the patients also needed bowel diversion due to intractable gastrointestinal morbidity, stressing the complexity of this disease [99].

Orthotopic reconstruction can be attempted to allow urethral voiding. This reconstruction involves cystoprostatectomy, ileal neobladder, and AUS placement. However, the scientific literature is scanty on outcomes of this option [42].

## 6. Post-Reconstructive Complications

Treatments of pelvic cancer, predominantly prostate cancer, can lead to important complications such as BMUS, PUS, and VUAS. Treatment of these complications can lead to even further and more severe complications. These potential post-reconstructive complications are de novo UI (up to 50%), new onset ED (up to 35%), fistulation, and any complication of perineal and intra-abdominal surgery, including vascular and septic complications. If UI occurs, an AUS can be safely implanted even in men after RT. Urethral reconstruction appears to have minimal impact on ED. Injury of the neurovascular bundle and cavernosal bodies occurs predominantly because of oncologic treatments.

## 7. Conclusions

Posterior urethral obstruction, and in its more severe form a devastated bladder outlet, is a frequent and challenging adverse event of pelvic malignancy management, especially prostate cancer, with significant morbidity and detriment to patient’s QoL. Cancer survivorship care is intrinsically associated with preservation of QoL through mitigation of adverse effects of successful oncological treatment. To state that these posterior urethral complications can be a reconstructive challenge is surely an understatement. Radiation therapy significantly increases a negative impact on treatment outcomes. Endoluminal interventions such as endoscopic incision with/without adjuvant intralesional injection of antifibrotic agents can be considered for all patients, including after RT, due to the high potential for complications resulting from open reconstruction. In select, refractory cases, successful reconstruction with durable outcomes is feasible even for challenging RT-induced stenoses and these men should be referred to high-volume institutions with expert surgeons for treatment. Nonetheless, urinary diversion with/without extirpative surgery is indicated as a last resort when reconstruction is unfeasible or futile such as in unmotivated patients not accepting a high complication rate, in the presence of unfavorable local anatomy making reconstruction extremely difficult or impossible or in the presence of a dysfunctional bladder and a necrotic, calcified, or densely scarred bladder outlet/posterior urethra. These patients are often satisfied with the outcomes of this last resort option.

## Figures and Tables

**Table 1:** Potential risk factors of postoperative posterior urethral obstruction.

Type of Risk Factor
Operative factors
Excessive blood loss
Persistent post-op urinary extravasation
Previous bladder outlet procedures
Surgeon’s experience
Surgical technique
Longer operating time
Type of anastomosis and suture
Migrated foreign body (e.g., hem-o-lock)
Duration of catheterization
Patient factors
Age
Cigarette smoking
BMI
Coronary artery disease
Hypertension
DM
Detrusor underactivity
Radiation therapy (adjuvant or salvage)

BMI = body mass index; DM = diabetes mellitus.

**Table 2:** Incidence of posterior urethral obstruction following primary treatment of PCa.

Treatment Modality	CaPSURE [5] % Stensosis (N. of pts)	Literature Review Range
RP RP + EBRT EBRT BT BT + EBRT Cryoablation HIFU Total	8.4% (3310) 2.7% (73) 1.7% (645) 1.8% (799) 5.2% (231) 2.5% (199) N/A 5.2% (6597)	1.6–29.9% [13,15,16,17,18,20,21,22] 2.7–10% [17,18,22] 2.0–13% [23,24] 0–14% [19,22,25] 1–32% [18,19] 1.1–3.3% [19,25] 1–31% [19,26,27,28]

PCa = prostate cancer; RP = radical prostratectomy; EBRT = external beam radiotherapy; BT = brachytherapy; Cryo = cryoablation; HIFU = high intensity focused ultrasound (Modified from Browne MB et al. [14]).

## Data Availability

This study is a narrative review.

## Acronyms

AUS	Artificial urinary sphincter
BMI	Body mass index
BMUS	Bulbomembranous urethral stenosis
BNS	Bladder neck stenosis
BOO	Bladder outlet obstruction
BT	brachytherapy
DVIU	Direct vision internal urethrotomy
EBRT	External beam radiotherapy
3D-CRT	Three-dimensional conformal radiation therapy
ED	Erectile dysfunction
EPA	Excision and primary anastomosis
HIFU	High intensity focused ultrasound
ICUD	International consultation of urologic disease
IMRT	Intensity modulated radiation therapy
MMC	Mitomycin C
PUS	Posterior urethral stenosis
QoL	Quality of life
RP	Radical prostatectomy
RALP	Robot-assisted laparoscopic prostatectomy
ROS	Reactive oxygen species
RRP	Radical retropubic prostatectomy
RT	Radiation therapy
SIU	Société International d’Urologie
TURP	Transurethral resection of prostate
UI	Urinary incontinence
UTI	Urinary tract infection
VUAS	Vesicourethral anastomotic stenosis
